# Development, implementation and evaluation of a bronchoscopy simulation training program for intensive care Fellows and intensivists in the Netherlands

**DOI:** 10.1177/0310057X251337756

**Published:** 2025-07-04

**Authors:** Eveline CF Gerretsen, Ulrich Strauch, Marleen Groenier, Walther NKA van Mook, Frank WJM Smeenk, Ruud PJ Segers

**Affiliations:** 1 Department of Educational Development and Research, School of Health Professions Education, Maastricht University, Maastricht, Netherlands; 2 Simulation Centre of Maastricht University Medical Centre+, Maastricht, Netherlands; 3 Technical Medical Centre, University of Twente, Enschede, Netherlands; 4 Department of Intensive Care Medicine, Maastricht University Medical Centre+, Maastricht, Netherlands; 5 Academy for Postgraduate Training, Maastricht University Medical Centre+, Maastricht, Netherlands; 6 School of Health Professions Education, Maastricht University, Maastricht, Netherlands; 7 Department of Respiratory Medicine, Catharina Hospital, Eindhoven, Netherlands

**Keywords:** Bronchoscopy, intensive care, simulation training

## Abstract

Simulation-based training can be valuable for teaching bronchoscopy to intensivists, providing a risk-free training environment. We developed, implemented and evaluated a simulation-based flexible bronchoscopy training program for intensive care Fellows and intensivists. This paper presents the development of its design and lessons learned. We used the Analyse, Design, Develop, Implement and Evaluate model for developing and evaluating the training program (Analysis and Design – phase 1, Development – phase 2, Implementation – phase 3, Evaluation – phase 4). In phase 1, two intensivists formulated learning objectives for bronchoscopy in an intensive care setting, which guided the identification and development of training materials and the preliminary training program (phase 2). In phase 3, we tested this program and gathered feedback from participants to guide program modifications. After implementing the adjusted training, we measured participants’ satisfaction using a survey based on closed- and open-ended questions (phase 4). Fifty-seven participants attended the training, with 18 (32%) responding to the questionnaire. Respondents highly appreciated the training program, with median satisfaction scores of 4 or higher on a five-point scale for all closed-ended questions. Respondents appreciated the supervision and feedback and found the simulator equipment relevant for learning bronchoscopy. This description of the program’s development and its evaluation results can serve as a valuable resource for those wishing to establish similar training programs. We recognise that further implementation of evidence-based instructional design principles might enhance the training program’s scientific foundation and effectiveness. We therefore recommend a more evidence-based approach for the design of future bronchoscopy simulation training programs.

## Introduction

Bronchoscopy procedures are commonly performed in the intensive care unit (ICU) for both diagnostic and therapeutic purposes.^1^ In rare cases, however, serious and potentially life-threatening complications can occur.^2^ The risk of such complications is higher in critically ill ICU patients.^3^ In the Netherlands, as in many European countries, experienced pulmonologists are not routinely part of the ICU team and may not be immediately available when acute bronchoscopy is needed. To help meet this demand, it is important that intensivists, too, have the skills to perform bronchoscopy in acute settings. This calls for adequate training of ICU specialists. As with many other procedural skills, the initial phase of such bronchoscopy skills training should ideally be simulation based. Simulation-based training (SBT) has shown to positively affect patient safety, the learner’s learning curve and the additional costs associated with occupying an operating theatre during a learning situation.^4,5^ Moreover, learning a procedure on patients when a simulation model is available is ethically questionable.^6^ For these reasons, SBT is to be preferred to non-simulation-based training in an apprenticeship model.

Several studies have investigated the use and effects of simulation-based flexible bronchoscopy training.^7–9^ To our knowledge, however, all training programs hitherto described were specifically designed for pulmonologists. In the Netherlands, intensivists have different roles in clinical practice and, consequently, they have also received different training with less or no focus on bronchoscopy skills. We therefore sought to develop a simulation training program specifically for intensive care Fellows and intensivists but also for other physicians working in ICU, to evaluate participant satisfaction following implementation and share lessons learned with the intensivist community.

## Materials and methods

We first defined the learning objectives for a simulation-based bronchoscopy training program in a critical care setting. Based on these objectives, we designed a preliminary training program which we first tested and subsequently adapted based on participant feedback. We then implemented the revised program and measured participant satisfaction afterwards to evaluate the training program, identifying training components that were most valued in the process.

## The Analyse, Design, Develop, Implement and Evaluate (ADDIE) model

**Figure 1.**
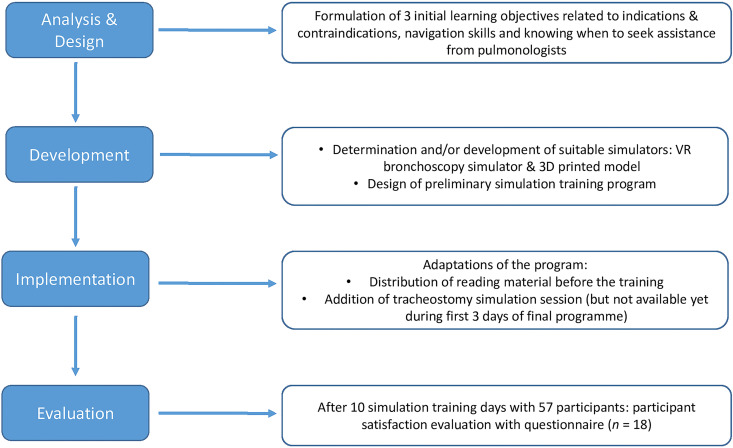
ADDIE model flowchart. ADDIE: Analyse, Design, Develop, Implement and Evaluate.

We employed the ADDIE model to retrospectively describe the process of developing, implementing and evaluating the bronchoscopy training program. This model is widely used by instructional designers to develop courses and training programs.^10,11^ Figure 1 shows a flowchart with a brief description of each of the four ADDIE phases as described in this article, but in the following, we will describe each phase in more detail.

### Phase 1: analysis and design

In 2018, two intensivists (RPJS and US) from Maastricht University Medical Centre+ (MUMC+), one of whom is also a pulmonologist, engaged in discussions about developing a bronchoscopy training program for intensivists. They both recognised the widespread use of bronchoscopy in the ICU and the need for intensivists to acquire bronchoscopy skills as experienced pulmonologists were not always available. If intensivists were trained to perform bronchoscopy in the ICU, this would improve the care of critically ill patients. The primary aim of the intended training program was to teach intensive care Fellows and intensivists basic bronchoscopy skills. However, it was decided to make the course available for other physicians also working in the ICU if space was available. Consensus was that the program should focus on the cornerstones of bronchoscopy, that is, knowledge of its indications, its technique and bronchial anatomy.^12^ Therefore, the first two learning objectives were: 1) being able to identify appropriate indications and relative contraindications for performing bronchoscopy in the ICU, and 2) being able to navigate the bronchial tree adequately (without unnecessary bronchial wall contact and with proper hand–eye coordination) while recognising the different lobes. Pulmonologists were invited to provide feedback on the learning objectives, which was fairly positive. However, they also emphasised that recognising situations requiring the expertise of a pulmonologist should be a key learning outcome of the training, leading to a final third learning objective.

### Phase 2: development of training materials

**Figure 2.**
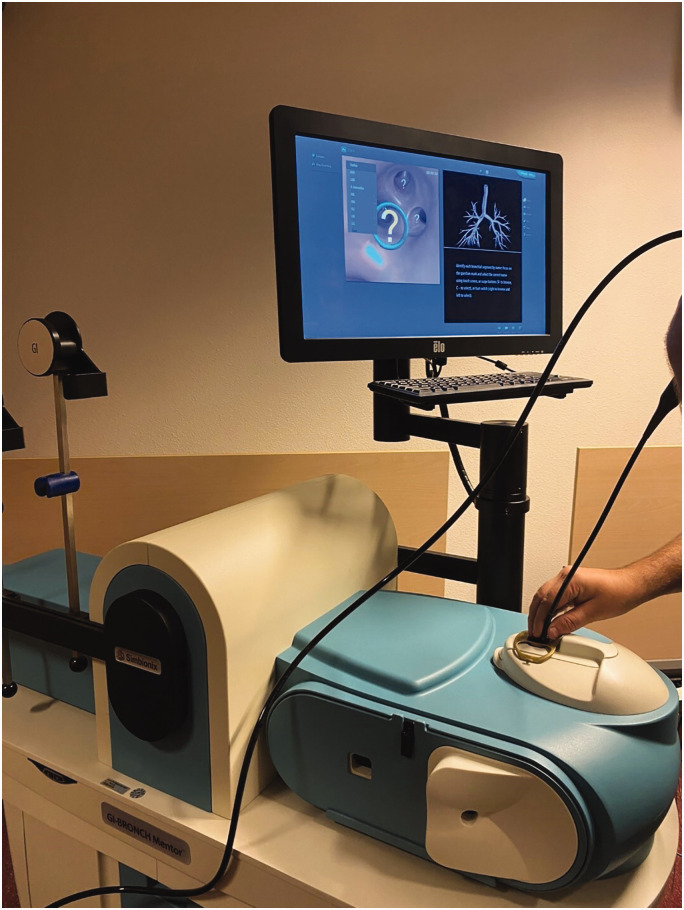
The Simbionix BRONCH Mentor flexible bronchoscopy simulator with the lung anatomy and bronchial segments task from the Essential Bronchoscopy module.

**Figure 3.**
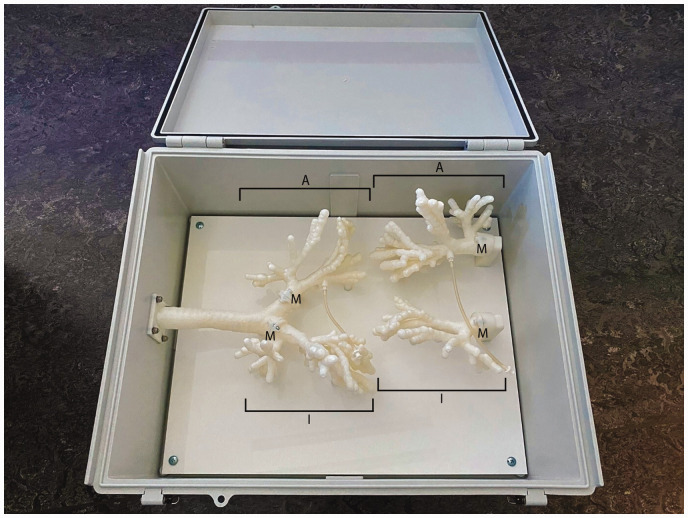
The three-dimensional-printed anatomical model developed by the Instrument Development, Engineering and Evaluation department. The box contains two lung models, each consisting of two parts. One part is suitable for inspection bronchoscopy only (depicted as I in the Figure), while the part with the intrabronchial line (depicted as A in the figure) can also be used to infuse the model with coloured soap to mimic blood or sputum, making it suitable for the simulation of aspiration of secretions in the endobronchial tree. Each part can be attached to and detached from the trachea by means of small magnets (depicted by M in the Figure).

A flexible virtual reality bronchoscopy simulator (Simbionix BRONCH Mentor, SurgicalScience, Sweden) (see Figure 2) was already available in the MUMC+ simulation centre. This simulator allowed trainees to practise their bronchoscopy skills at different levels, ranging from basic navigation skills to actual diagnostic and therapeutic patient cases. To enhance the fidelity of the learning experience and to achieve the learning objectives, the training developers collaborated with the Instrument Development, Engineering and Evaluation (IDEE) department at Maastricht University to create an anatomically accurate model for training navigation skills and interventions such as sputum aspiration. Aided by a thoracic radiologist, the IDEE department eventually constructed a three-dimensional (3D)-printed model of a bronchial tree, based on a thoracic computed tomography scan, that could also be used for aspiration of fluids (see Figure 3).

Based on existing literature and the learning objectives and training materials thus obtained, we went on to design a preliminary simulation training program that lasted one day (see Table 1). In the session with the virtual reality simulator, participants started with some basic simulation tasks to familiarise themselves with navigating the scope, and then, if their skill level allowed, moved to more complex diagnostic and therapeutic patient cases. The session with the 3D-printed anatomical model comprised practising navigation skills and aspiration interventions, using red soap infused in the model to mimic blood. Instruction was provided by the training developers, both with extensive experience in performing clinical bronchoscopy and in training residents, for which they followed the mandatory train-the-trainer courses at the MUMC+ on how to give meaningful feedback to residents in the workplace. Each session could accommodate three participants at a time, with the groups rotating after finishing their session.

**Table 1.** Content of the preliminary and final simulation training program.

Activity	Subject/description (duration)	Preliminary	Final
Reading materials	The Dutch Thoracic Society outline for bronchoscopy in ICUs (N/A)Links to bronchoscopy websites (N/A)	Available during the training	Distributed beforehand
Presentation	Indications, contraindications and preparations for bronchoscopy (by pulmonologists) (45 min)	+	+
Presentation	Bronchial/segmental anatomy (30 min)	+	+
Presentation	Clinical cases (45 min)	+	+
Presentation	The bronchoscope (30 min)	+	+
Simulation session 1	Simbionix simulator exercises (30 or 60 min)	+60 min	+Session 1 and 2 combined: 120 min on days 1–3,60 min on days 4–10
Simulation session 2	Practical exercises (aspiration interventions) with the 3D-printed bronchial anatomical model (30 or 60 min)	+60 min	
Simulation session 3	Tracheostomy simulation procedure (60 min)	–	+Available only on days 4–10
Verbal evaluation	Participants are invited to provide verbal feedback and evaluate the training program (15 min)	+	+

N/A = not applicable as no allocated duration was made for this activity; + = included in the training program; – = not included in the training program.

ICU: intensive care unit; 3D: three-dimensional.

### Phase 3: implementation of the simulation training program and its further development

**Figure 4.**
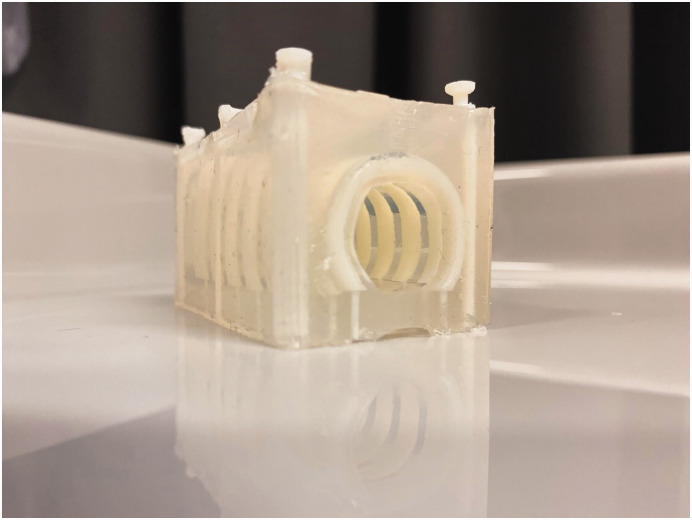
The three-dimensional-printed tracheostomy training model that can be inserted into a training manikin.

The training developers piloted the preliminary program on colleagues from the ICU department during three separate days. Based on the feedback obtained, we made several final changes to the program. First, we ensured that participants received the reading materials a few weeks ahead of the training to give them the opportunity to prepare and optimise learning outcomes.^13–15^ Second, the participants in this pilot recommended adding a simulation session to the program to practise using the bronchoscope during a percutaneous tracheostomy procedure, which is a common intervention in most ICUs. Consequently, the IDEE department was asked to develop a realistic 3D-printed tracheostomy training model that could be inserted into a manikin (the Advanced HAL S3201, developed by Gaumard, Miami, Florida, USA). This manikin features a pre-existing tracheal opening, equipped with a standard trachea model. However, the standard model could not be used to simulate a complete tracheostomy procedure owing to the texture of the material. The new model developed by the IDEE department (see Figure 4) allows trainees to practise a complete and realistic percutaneous tracheostomy procedure under bronchoscopic guidance.

The tracheostomy simulation procedure involved identifying anatomical landmarks, puncturing the trachea under bronchoscopic guidance, introducing the guide-wire and dilating the trachea with small and larger dilators. Finally, the trachea cannula was introduced, and the bronchoscope was used to confirm its correct placement.

After these training program adaptations, we implemented the final program (see Table 1) in 10 separate runs at the MUMC+ simulation centre in the period spanning 6 June 2019 to 8 April 2022. The training program was non-mandatory, and participants could voluntarily participate based on their interest and how well it aligned with their clinical responsibilities. The program was open to healthcare professionals from various hospitals across the southern region of the Netherlands. To ensure optimal interaction and a safe learning environment, each training day accommodated a maximum of six participants. The tracheostomy training model was not available until February 2020 and was therefore used only in the last seven training days. Once the model was available, the sessions with the virtual reality simulator and the 3D-printed anatomical model were combined. This arrangement allowed three participants to join the tracheostomy simulation session, while the other three alternated between the virtual reality simulator and the 3D anatomy model. This approach ensured no extra instructors were needed and kept group sizes small. No additional program changes were made.

### Phase 4: evaluation—participants’ satisfaction

To assess the perceived relevance of the simulation training and participants’ satisfaction with its contents, materials, and supervision quality we developed a questionnaire. This questionnaire comprised open-ended questions and closed-ended questions or statements to be rated on a five-point Likert scale (1 = strongly disagree, 2 = disagree, 3 = neither agree nor disagree, 4 = agree, 5 = strongly agree). Drawing from several papers addressing student satisfaction with bronchoscopy SBT or simulation-based learning in general,^16–20^ the first author created a first draft of the questionnaire, with seven open-ended questions and 19 closed-ended statements. Four of the other authors consequently reviewed this initial draft (RPJS, MG, WNKAvM, FWJMS) in two separate rounds. This step led to the addition of four open-ended questions and six statements, the omission of five statements, and minor adaptations to five statements. The final questionnaire (see Supplemental material file 1 online), with 11 open-ended and 20 closed-ended statements, was then sent out to all participants. All open-ended questions were mandatory, ensuring responses from all participants. The qualitative data from the open-ended questions were analysed for common themes by the first and last author. After independent scrutiny, the two authors held a consensus meeting to compare the results from their individual analyses and resolve any discrepancies. In the case of discrepancies being unable to be resolved, the team as a whole discussed the matter and took a final decision.

Survey results were analysed using IBM SPSS statistics (version 28; IBM Corp, Armonk, NY, USA). Given the non-normal distribution of the statement results, differences between the participant categories were compared with the Kruskal–Wallis test. Bonferroni’s correction resulted in a corrected threshold of α = 0.0025.

## Ethics

This study was carried out in accordance with the Declaration of Helsinki. Completing the survey was voluntary and entirely anonymous. Before filling in the questionnaire, participants gave their informed consent by completing the designated form they had received together with the questionnaire link. Dutch law stipulates that ethics approval is needed only when a study falls under the Medical Research Involving Human Subjects Act. As an anonymous, short questionnaire that does not contain any sensitive questions, our survey did not fall under this act.^21,22^ Consequently, it did not qualify for review by an institutional ethics committee.

## Results

All training days were quickly fully booked, indicating a high level of interest. The implementation of the adapted training proceeded without any issues. The training days were attended by a total of 57 participants, predominantly intensive care Fellows and intensivists (*n* = 45; see Table 2). In this context, the term ‘intensive care Fellows’ refers to physicians undergoing super- or intra-specialty training in intensive care medicine (a total duration of two years in the Netherlands, following their initial specialisation in fields such as internal medicine or anaesthesiology), while ‘intensivists’ refers to former Fellows who have completed this intensive care training. Some other physicians, who work only temporarily in intensive care, also participated in the training program upon special request and when space was available. These participants will hereinafter be referred to as non-ICU physicians. Of all participants, 18 (hereinafter referred to as respondents) completed the questionnaire (response rate 32%). Among these respondents, seven were intensivists, seven were intensive care Fellows and four were non-ICU physicians when they participated in the training.

**Table 2.** Background of training participants.

Participant category	Primarily (being) trained as	Number of participants
Intensivists	Internist	23
	Anaesthesiologist	5
	Cardiologist	2
	Surgeon	1
Fellow intensivists	Anaesthesiologist	7
	Internist	4
	Cardiologist	2
	Surgeon	1
Residents	Pulmonologist	3
	Anaesthesiologist	1
Non-ICU physicians	Physician assistant	5
	Anaesthesiologist	1
	Emergency physician	1
	Foundation physician	1

ICU: intensive care unit.

### Participant satisfaction

Table 3 presents participants’ median ratings of the questionnaire statements for all respondents and per respondent category. In cases where the virtual reality simulator was not operational owing to a technical defect or when the tracheostomy training model was not yet available, participants did not score the related statements. While all but two statements received high median agreement scores of at least 4 across all respondent categories, indicating overall satisfaction with the training, there appeared to be a slight but not significant tendency for intensivists to rate some statements lower.

**Table 3.** Respondents’ questionnaire ratings of each item on a five-point Likert scale.

Statement category	Statement	All*N* = 18Median (IQR)	Intensivist*n* = 7Median (IQR)	Fellow intensivistsn = 7Median (IQR)	Non-ICU*n* = 4Median (IQR)
General	I had sufficient time to practise	4 (4–5)	4 (3–4.5)	4 (4–5)	5 (4–N/A^a^)
	The training made me feel more competent to perform future bronchoscopies	5 (4–5)	4 (4–4.5)	5 (4–5)	5 (5–5)
	The training prepared me well for performing future bronchoscopies in the intensive care suite	4 (4–5)	4 (4–4.5)	4 (4–5)	5 (5–5)
	If an intensivist wishes to receive bronchoscopy training, part of this training should be simulation based	5 (4–5)	5 (4–5)	4 (4–5)	5 (5–5)
	On a scale of 1 to 5, how satisfied are you with the training?	4.5 (4–5)	4 (4–5)	4.5 (4–5)	5 (4–N/A^a^)
Training materials	The reading materials offered were relevant	4 (4–5)	4 (3–4)	4 (3.8–5)	5 (5–5)
	The content of the presentations was relevant	4 (4–5)	4 (4–4)	4 (4–5)	5 (5–5)
	The content of the presentations was clear	4 (4–5)	4 (4–4)	4 (4–5)	5 (5–5)
	The presenting style of the presentations was clear	4 (4–5)	4 (4–4)	4 (4–5)	5 (5–5)
VR simulator	Operating the bronchoscope of the VR simulator was realistic	4 (4–5)	4 (4–4.5)	4.5 (4–5)	4 (3–N/A^a^)
	The anatomy depicted in the VR simulator was realistic	4 (4–5)	4 (4–4.5)	4.5 (4–5)	5 (4–N/A^a^)
	The VR simulator was relevant to learning to perform an inspection bronchoscopy	4 (4–5)	4 (4–4.5)	4.5 (4–5)	5 (4–N/A^a^)
3D bronchoscopy model	The anatomy of the 3D bronchoscopy model was realistic	4.5 (4–5)	4 (3–5)	4.5 (4–5)	5 (5–5)
	The 3D bronchoscopy model was relevant to learning to perform an inspection bronchoscopy	4 (4–5)	4 (3–5)	4 (4–5)	5 (5–5)
Simulated tracheostomy procedure	The simulated tracheostomy procedure was realistic	4 (4–5)	4 (4–4)	4 (3.8–5)	5 (4–N/A^a^)
	The simulated tracheostomy procedure was relevant to learning to perform a tracheostomy procedure under bronchoscopic guidance	4 (4–5)	4 (4–4)	4 (4–5)	5 (4–N/A^a^)
Competences	The training improved my bronchoscopy competences	4 (4–5)	4 (3.5–4.5)	4 (4–5)	5 (5–5)
	The training made me able to inspect all lobes and segments	4 (3.8–5)	3 (2.5–4.5)	4 (4–5)	5 (5–5)
Supervision	I am satisfied with the supervision received	5 (4–5)	4 (4–4.5)	5 (4–5)	5 (5–5)
	I am satisfied with the level of expertise of the instructors	5 (4–5)	4 (4–4.5)	5 (4–5)	5 (5–5)

Likert scale: 1 = strongly disagree, 2 = disagree, 3 = neither agree nor disagree, 4 = agree, 5 = strongly agree.

Three participants could not answer the questions about the VR simulator because it was not operational due to a technical defect, and one participant could not answer the questions about the tracheostomy model as it was not yet available.

a N/A = not applicable as the 75th percentile could not be calculated in some cases owing to the limited sample size of the non-ICU group.

IQR: interquartile range; VR: virtual reality; ICU: intensive care unit.

### Open-ended questions

Analysis of the qualitative data from the open-ended questions revealed the following common themes:

#### 1. Good balance between theory and practice

Respondents appreciated the well-balanced combination of theory and practice in the training, as evidenced by the following statements:

‘[I liked] the alternation between practice and theory. Good basic information, applied to clinical cases. A lot of opportunities to practise on the training models with direct feedback.’

‘Good build-up from theory to practice. The hands-on training was enlightening.’

#### 2. Participants were satisfied with supervision

Respondents expressed satisfaction with the supervision provided during the training. They especially flagged the feedback from the simulator and support from the instructors as positives, as the following answers to the question ‘What did you like about the training?’ demonstrate:

‘[I liked] the supervision, that we got feedback from the simulator and good support from instructors.’

‘[I liked the] good supervision … and good support from the instructors.’

#### 3. Increased confidence and competence

Respondents reported feeling more confident and competent to perform bronchoscopy in an intensive care setting as a result of the training. In their words:

‘[…] The anatomy is much clearer now. I feel more competent to perform a bronchoscopy.’

‘I am more confident in performing the procedure.’

#### 4. The training is recommendable

All respondents stated that they would recommend the training to others, as the following answers demonstrate:

‘Yes [I would recommend the training to others], I think everyone working in the ICU team should have participated in this course.’

‘Yes [I would recommend the training to others], it was a good hands-on training program for non-pulmonologists who would like to perform bronchoscopies in the ICU.’

#### 5. Positive perception of simulators

Respondents expressed positive views about the simulators used in the training. They considered the virtual reality simulator as relevant and enjoyable for practice, because of its built-in game-like tasks. Similarly, the 3D bronchoscopy model was perceived as relevant, as it allowed participants to practise interventions. The same held true for the tracheostomy simulation procedure. The following statements illustrate these positive perceptions:

‘[I liked] the content, structure and especially the simulation part of the training.’

‘[The virtual reality simulator] was fun, very informative and challenging.’

‘[The 3D-printed bronchoscopy model is] convenient to work with. You can see what you are doing.’

‘I liked it [the tracheostomy simulation procedure]. [It was a] realistic way of practising. [The] simulation equipment [was] good. We practised the entire procedure.’

#### 6. No clear simulator preference

Participants’ responses did not point to a specific preference for a simulator when practising inspection bronchoscopy. While some respondents (*n* = 6) equally liked both the 3D-printed bronchoscopy model and the virtual reality simulator, others expressed a preference for either one. Those who preferred the virtual reality simulator (*n* = 5) appreciated the immediate feedback it offered, whereas those who favoured the 3D-printed bronchoscopy model (*n* = 4) perceived it as more realistic.

#### 7. Desire for additional practice and skill refresher opportunities

As points of improvement, respondents especially expressed a desire for more practice time and the opportunity to re-engage with parts of the training at a later time for skill enforcement. This desire was demonstrated by the answers to the question ‘Do you see any opportunities for improving the training?’:

‘To have the option for revisiting the training at a later time.’

‘More practice is always better.’

‘For the retention of [the acquired skills], it would be beneficial to repeat the training after two months.’

## Discussion

In this article, we described the development, implementation and evaluation of a one-day simulation-based bronchoscopy training program for trainees and specialists working in an intensive care setting. To our knowledge, this study was the first describing the design of such a program specifically for intensivists. Evaluation outcomes demonstrated that participants were very satisfied with the training, as all questionnaire statements received a median score of at least 4 out of 5. The answers to the open-ended questions furthermore revealed that participants appreciated the supervision they received as well as the feedback from both simulators and instructors. They also valued the simulator equipment which they perceived as relevant. Finally, participants confirmed that they would recommend the training to colleagues.

Although no significant differences were observed in the survey results between the different participant categories, there appeared to be a trend towards intensivists rating statements somewhat lower compared with intensive care Fellows and other physicians working in the ICU. This could likely be attributed to their past experiences with bronchoscopy procedures in the ICU, suggesting that the training program in its current form may be the most beneficial for physicians with less bronchoscopy experience. Therefore, we believe that tailoring the training content to different experience levels will most probably enhance overall participant satisfaction even more. This tailoring could be achieved by gauging participants’ experience levels at the start of the training or by organising separate training days for different experience levels.

The fact that participants appreciated the feedback from both simulators and instructors emphasises the importance of keeping training groups small so as to create a safe learning environment in which participants have ample opportunity to practise. This reiterates prior qualitative research findings that limiting the number of participants in simulation-based bronchoscopy training contributes to a safe and positive learning environment.^23^ Specifically, a group size of four is recommended, with a maximum of six participants. In our study, groups in the simulation sessions were even smaller, with three participants, which allowed for even more practice time than the recommended group size. Our evaluation outcomes also showed that participants favoured simulators that were both fun (containing playful learning or serious gaming) and realistic. Yet, previous research in other medical professions has revealed that such high-fidelity simulators (i.e. simulators with a high degree of realism) do not necessarily contribute to procedural skill improvement.^24^ As only one study related to bronchoscopy was included in this review, we encourage researchers to examine the effects of simulator realism on skill transfer in the specific context of clinical bronchoscopy.

Despite the training program being valued by participants, we recognise some limitations. First, no formal instructional design theory was used when the training program was developed. The intensivists possessed a profound enthusiasm for the potential of simulation training to teach bronchoscopy skills to colleagues, but we recognise that certain fundamental principles of instructional design may not have been addressed, such as constructive alignment^25^ and a thorough needs assessment.^26^ Specifically, constructive alignment involves ensuring that all simulation activities are aligned with the learning objectives and that validated assessment methods (such as the Bronchoscopy Skills and Tasks Assessment Tool (BSTAT)^27^) are used that evaluate whether trainees have achieved these objectives. In our training program, while learning activities were aligned with the learning objectives, validated assessment methods to evaluate trainees’ skills and knowledge were lacking. Additionally, the preliminary training program was developed without a thorough needs assessment: it lacked a tracheostomy simulation session, which was later added to the final program based on feedback of colleagues. A robust needs assessment would involve identifying bronchoscopy skill and knowledge gaps among intensivists before designing the initial training program, by gathering input from intensivists and other stakeholders such as pulmonologists. This input should then inform the formulation of learning objectives. Using these instructional design principles from the start might have resulted in a training program aligning more with educational theory, probably leading to better transfer to clinical practice. Second, a critical aspect of any training program is the formulation of specific and measurable learning objectives. In our case, these were not explicitly defined and evaluated from the beginning. Together with the absence of objective, quantitative evaluation measures to measure skill improvement, we believe that this limited our ability to demonstrate true training effectiveness. Third, the relatively low response rate of 32% to the survey may have introduced non-response bias, which could have affected our final results.^28^ Additionally, it is worth noting that while a relatively large number of intensivists participated in the training (31 out of 57), only seven completed the questionnaire. This limited response rate among intensivists may have led to a small overestimation of the overall positive evaluation of the training program, as their feedback, although not statistically significant, seemed slightly less favourable compared with other participants. Despite this limitation, we believe the positive feedback from all participants who responded highlights the program’s potential. Finally, we did not pay any attention to the role of instructors in participant satisfaction with the training program. Investigating the impact of instructors on participant satisfaction might provide valuable insights for future study or program redesign.

In conclusion, our findings suggest that SBT can be a well-received tool to teach intensivists bronchoscopy skills. The program’s content could be further enhanced by incorporating instructional design principles during the analysis and design phase and by tailoring the program to learners’ needs. Furthermore, implementing a validated summative assessment such as the BSTAT and exploring the impact of instructors on the program’s success is highly recommended. We therefore recommend that intensivist educators design future bronchoscopy simulation training programs according to a more evidence-based approach. Nevertheless, we believe that our comprehensive description of the program’s development process and the evaluation results provided in this study can serve as a valuable resource for those wishing to establish similar training programs.
